# Supplementary material for: A genome-wide investigation of microsatellite mismatches and the association with body mass among bird species
Source: PeerJ. 2018 Mar 14;6:e4495. doi: 10.7717/peerj.4495 (PMC5857172; doi:10.7717/peerj.4495)
Supplement: Figure S1 [file peerj-06-4495-s001.pdf]

The script that performs the PGLS analyses in R

```
library (ape)

library (caper)

#load in trees

Tre=read.nexus("birdtree.nex")

#load in the data which are presented in the supplements of the paper

data <- read.csv("Table_S1.csv", header=T)

dat<-comparative.data(Tre, data, "Species")

#PGLS model

mod1<- pgls(1/Mismatch~log(Body_mass),dat, lambda="ML")

mod2<- pgls(1/Mismatch~log(Body_mass)+asin(sqrt(GC_content)),dat,
lambda="ML")

#summary of the models

summary(mod1,mod2)
```
