# Supplementary material for: A genome-wide investigation of microsatellite mismatches and the association with body mass among bird species
Source: PeerJ. 2018 Mar 14;6:e4495. doi: 10.7717/peerj.4495 (PMC5857172; doi:10.7717/peerj.4495)
Supplement: Figure S2 — The phylogeny is presented in Nexus format and can be drawn using any standard tree-drawing package such as TreeView. [file peerj-06-4495-s002.pdf]

```
#NEXUS
BEGIN TREES;
	TRANSLATE
		1	Picoides_pubescens,
		2	Merops_nubicus,
		3	Buceros_rhinoceros,
		4	Apaloderma_vittatum,
		5	Leptosomus_discolor,
		6	Colius_striatus,
		7	Tyto_alba,
		8	Haliaeetus_leucocephalus,
		9	Haliaeetus_albicilla,
		10	Cathartes_aura,
		11	Lonchura_striata,
		12	Taeniopygia_guttata,
		13	Passer_domesticus,
		14	Geospiza_fortis,
		15	Sturnus_vulgaris,
		16	Phylloscopus_trochilus,
		17	Zosterops_lateralis,
		18	Parus_major,
		19	Corvus_brachyrhynchos,
		20	Lepidothrix_coronata,
		21	Manacus_vitellinus,
		22	Acanthisitta_chloris,
		23	Agapornis_roseicollis,
		24	Amazona_aestiva,
		25	Nestor_notabilis,
		26	Melopsittacus_undulates,
		27	Falco_peregrinus,
		28	Cariama_cristata,
```

29 Pelecanus_crispus,

30 Egretta_garzetta,

31 Nipponia_nippon,

32 Phalacrocorax_carbo,

33 Pygoscelis_adeliae,

34 Aptenodytes_forsteri,

35 Fulmarus_glacialis,

36 Gavia_stellata,

37 Phaethon_lepturus,

38 Eurypyga_helias,

39 Uria_lomvia,

40 Charadrius_vociferous,

41 Grus_japonensis,

42 Balearica_regulorum,

43 Gallirallus_okinawae,

44 Opisthocomus_hoazin,

45 Chaetura_pelagica,

46 Calypte_anna,

47 Antrostomus_carolinensis,

48 Tauraco_erythrolophus,

49 Chlamydotis_macqueenii,

50 Cuculus_canorus,

51 Pterocles_gutturalis,

52 Mesitornis_unicolor,

53 Patagioenas_fasciata,

54 Columba_livia,

55 Podiceps_cristatus,

56 Phoenicopterus_rubber,

57 Gallus_gallus,

58 Meleagris_gallopavo,

59 Callipepla_squamata,

60 Numida_meleagris,

61 Anser_brachyrhynchus,

62 Anser_cygnoides,

63 Anas_platyrhynchos,

64 Struthio_camelus,

65 Tinamus_major

;

TREE * 65birds= (((((((((7:61.649200,(6:59.941898,(5:58.571899,(4:55.095600,(3:51.984901,(1:42.341801,2:42.341801):9.643100):3.110700):3.476300):1.370000):1.707300):1.122800,(10:60.349098,(8:5.992200,9:5.992200):54.356899):2.422900):0.703100,(28:62.102901,(27:59.992901,((22:38.477901,((19:20.525999,((15:11.824519,((11:6.839130,12:6.839130):3.076270,(13:6.714970,14:6.714970):3.200430):1.909119):1.994549,(18:12.449678,(16:1.897191,17:1.897191):10.552487):1.369390):6.706832):11.291000,(20:8.647782,21:8.647782):23.169218):6.660900):15.977700,((23:20.050646,24:20.050646):18.246998,(25:28.866301,26:28.866301):9.431343):16.157957):5.537300):2.110000):1.372300):1.968400,((36:62.800800,((32:58.922100,(31:57.558201,(29:55.242599,30:55.242599):2.315600):1.363900):2.429900,(35:59.986401,(33:22.998301,34:22.998301):36.987999):1.365700):1.448700):1.724500,(37:60.021000,38:60.021000):4.504300):0.918200):1.291100,(44:65.928596,((39:56.248158,40:56.248158):8.327242,(43:49.824833,(41:17.742760,42:17.742760):32.082073):14.750566):1.353200):0.806000):1.214800,((47:62.939602,(45:43.651402,46:43.651402):19.288200):3.798700,(50:64.898003,(48:62.256199,49:62.256199):2.641800):1.840300):1.211100):1.244000,(((51:61.612000,52:61.612000):3.996100,(53:23.883490,54:23.883490):41.724609):2.513200,(55:56.331799,56:56.331799):11.789500):1.072100):19.377899,((60:39.572037,(59:36.005951,(57:27.912201,58:27.912201):8.093750):3.566088):26.211163,(63:18.214306,(61:0.604714,62:0.604714):17.609591):47.568893):22.788099):13.067100,(64:83.763603,65:83.763603):17.874901);

ENDBLOCK;
