# Supplementary figures and images for: A genome-wide investigation of microsatellite mismatches and the association with body mass among bird species
Source: PeerJ. 2018 Mar 14;6:e4495. doi: 10.7717/peerj.4495 (PMC5857172; doi:10.7717/peerj.4495)

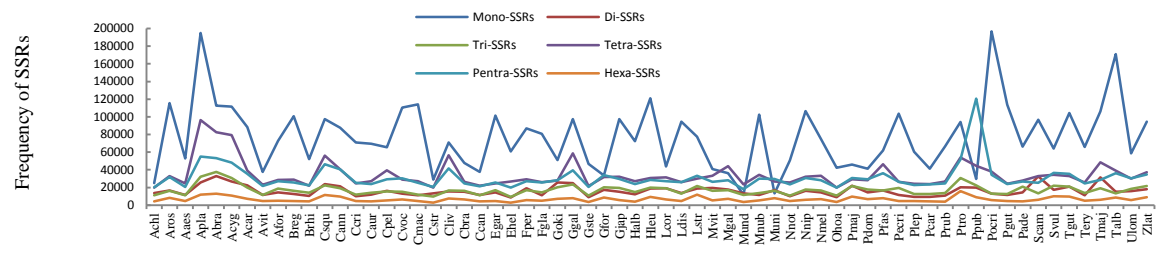

Supplement: Figure S3 [file peerj-06-4495-s003.pdf]

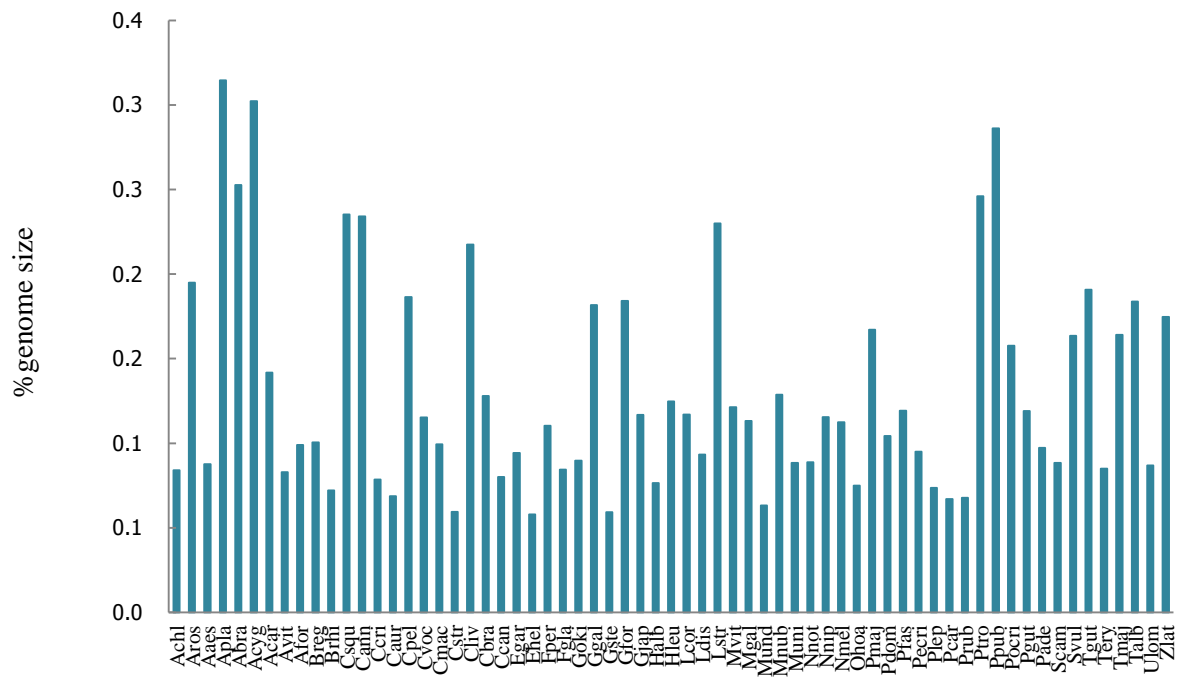

Supplement: Figure S4 — The abbreviated bird names are shown in the x-axis and the percentages are shown in the y-axis. [file peerj-06-4495-s004.pdf]
