# Supplementary material for: A genome-wide investigation of microsatellite mismatches and the association with body mass among bird species
Source: PeerJ. 2018 Mar 14;6:e4495. doi: 10.7717/peerj.4495 (PMC5857172; doi:10.7717/peerj.4495)
Supplement: Table S1 — Species names are abbreviated with four letters; first letter represents the genus name and last three letters represent the species name. For Pelecanus crispus and Podiceps cristatus, we use Pecri and Pocri separately. Mono-, di-, tri-, tetra-, penta- and hexa- are microsatellite types. [file peerj-06-4495-s005.docx]

**Table S1: A list of the 65 avian species and average adult body mass, GC content, average mismatches of imperfect microsatellites on the whole genome.** Species names are abbreviated with four letters; first letter represents the genus name and last three letters represent the species name. For *Pelecanus crispus* and *Podiceps cristatus*, we use Pecri and Pocri separately. Mono-, di-, tri-, tetra-, penta- and hexa- are microsatellite types.

| **Species** | **Abbreviated**  **Species names** | **Body mass(g)** | **GC content(%)** | **Average Mismatches of imperfect microsatellites** | | | | | |
| --- | --- | --- | --- | --- | --- | --- | --- | --- | --- |
|  |  |  |  | **All** | **Di-** | **Tri-** | **Tetra-** | **Penta-** | **Hexa-** |
| *Acanthisitta chloris* | **Achl** | 6.91 | 41.6 | 2.08 | 1.92 | 1.96 | 2.13 | 2.1 | 3.96 |
| *Agapornis roseicollis* | **Aros** | 53.83 | 41 | 2.21 | 2.28 | 2.02 | 2.55 | 2.3 | 3.38 |
| *Amazona aestiva* | **Aaes** | 451 | 42.2 | 1.81 | 1.87 | 1.67 | 1.87 | 1.92 | 2.8 |
| *Anas platyrhynchos* | **Apla** | 843.42 | 41.2001 | 1.81 | 1.79 | 1.82 | 1.68 | 1.71 | 2.69 |
| *Anser brachyrhynchus* | **Abra** | 2642.04 | 41.1 | 1.83 | 2.09 | 1.79 | 1.72 | 1.91 | 2.52 |
| *Anser cygnoides* | **Acyg** | 3511.94 | 41.5 | 2.4 | 2.12 | 1.91 | 2.19 | 3.77 | 5.37 |
| *Antrostomus carolinensis* | **Acar** | 109 | 40.8 | 1.98 | 1.87 | 2.06 | 1.91 | 1.92 | 3.55 |
| *Apaloderma vittatum* | **Avit** | 55 | 41.4 | 2 | 1.87 | 2.02 | 2.34 | 1.98 | 3.62 |
| *Aptenodytes forsteri* | **Afor** | 34394 | 42 | 1.98 | 1.75 | 2.05 | 2.15 | 2.38 | 2.86 |
| *Balearica regulorum* | **Breg** | 3771.99 | 41.2001 | 1.73 | 1.79 | 1.66 | 1.78 | 1.86 | 2.8 |
| *Buceros rhinoceros* | **Brhi** | 2371.58 | 42.6 | 1.85 | 1.85 | 1.7 | 2.25 | 1.98 | 2.96 |
| *Callipepla squamata* | **Csqu** | 183.86 | 40.6 | 2.09 | 2.13 | 1.87 | 1.99 | 2.32 | 4.1 |
| *Calypte anna* | **Cann** | 4.24 | 41.3 | 2.66 | 2.84 | 2.55 | 3.05 | 2.71 | 4.34 |
| *Cariama cristata* | **Ccri** | 1400 | 41.2 | 1.68 | 1.51 | 1.56 | 1.81 | 1.86 | 2.64 |
| *Cathartes aura* | **Caur** | 1518.24 | 41.1 | 1.62 | 1.57 | 1.75 | 1.79 | 1.7 | 2.48 |
| *Chaetura pelagica* | **Cpel** | 23.6 | 41.6 | 2.86 | 2.93 | 2.59 | 3.86 | 2.88 | 4.16 |
| *Charadrius vociferous* | **Cvoc** | 96.44 | 42 | 1.87 | 1.9 | 2.04 | 2.1 | 2.04 | 3.41 |
| *Chlamydotis macqueenii* | **Cmac** | 1501.33 | 41.1 | 1.66 | 1.49 | 1.57 | 1.85 | 1.68 | 2.61 |
| *Colius striatus* | **Cstr** | 51.1 | 40.9 | 1.9 | 1.94 | 1.8 | 2.1 | 1.89 | 3.52 |
| *Columba livia* | **Cliv** | 354.2 | 41.6 | 2.62 | 2.08 | 2.32 | 3.44 | 2.17 | 4.29 |
| *Corvus brachyrhynchos* | **Cbra** | 448.76 | 42 | 2.48 | 2.17 | 2.14 | 3 | 2.61 | 4.36 |
| *Cuculus canorus* | **Ccan** | 111.36 | 41.7 | 2.14 | 2.33 | 2.15 | 2.31 | 2.42 | 3.2 |
| *Egretta garzetta* | **Egar** | 312 | 42.5 | 1.73 | 1.62 | 1.67 | 2.01 | 1.97 | 3.11 |
| *Eurypyga helias* | **Ehel** | 210 | 42.3 | 1.72 | 1.67 | 1.64 | 1.94 | 1.81 | 2.6 |
| *Falco peregrinus* | **Fper** | 722.4 | 41.8 | 1.95 | 2.05 | 1.76 | 2.14 | 2.12 | 3.18 |
| *Fulmarus glacialis* | **Fgla** | 611.94 | 41.2 | 1.77 | 1.58 | 1.67 | 1.92 | 1.85 | 2.63 |
| *Gallirallus okinawae* | **Goki** | 433 | 42.7 | 1.76 | 1.63 | 2.08 | 1.78 | 1.67 | 2.43 |
| *Gallus gallus* | **Ggal** | 751.72 | 41.9346 | 1.99 | 2.02 | 1.6 | 2.06 | 1.99 | 3.25 |
| *Gavia stellata* | **Gste** | 1486 | 41.1001 | 1.8 | 1.77 | 2.1 | 1.9 | 1.75 | 3 |
| *Geospiza fortis* | **Gfor** | 24 | 41.7 | 2.66 | 2.45 | 2.28 | 3.33 | 2.47 | 4.15 |
| *Grus japonensis* | **Gjap** | 8785.99 | 41.6 | 1.91 | 1.72 | 1.65 | 1.66 | 1.78 | 2.3 |
| *Haliaeetus albicilla* | **Halb** | 4729.27 | 40.9 | 1.69 | 1.44 | 1.94 | 1.77 | 1.77 | 2.59 |
| *Haliaeetus leucocephalus* | **Hleu** | 4700.58 | 41.8 | 2.15 | 1.58 | 1.99 | 1.81 | 1.89 | 5.1 |
| *Lepidothrix coronata* | **Lcor** | 8.34 | 41.9 | 2.05 | 2.22 | 1.83 | 2.3 | 2.05 | 3.19 |
| *Leptosomus discolor* | **Ldis** | 255 | 41.8 | 1.81 | 1.71 | 1.71 | 2.15 | 1.84 | 2.99 |
| *Lonchura striata* | **Lstr** | 12.3 | 41.8 | 2.4 | 2.49 | 2.43 | 2.56 | 2.28 | 4.16 |
| *Manacus vitellinus* | **Mvit** | 18.15 | 41.2 | 2.22 | 2.31 | 2.26 | 2.27 | 2.4 | 3.72 |
| *Meleagris gallopavo* | **Mgal** | 5791.37 | 41.7219 | 1.77 | 1.96 | 1.68 | 1.67 | 1.77 | 2.27 |
| *Melopsittacus undulates* | **Mund** | 29.1 | 41.4001 | 1.85 | 1.83 | 1.76 | 1.86 | 1.96 | 3.01 |
| *Merops nubicus* | **Mnub** | 42.4 | 41.7 | 1.86 | 1.9 | 1.99 | 2.07 | 2.02 | 3.13 |
| *Mesitornis unicolor* | **Muni** | 148 | 41.3 | 2.07 | 2.05 | 1.97 | 2.02 | 1.92 | 3.22 |
| *Nestor notabilis* | **Nnot** | 862.97 | 41.1 | 1.84 | 1.63 | 1.7 | 2.09 | 1.96 | 2.79 |
| *Nipponia nippon* | **Nnip** | 1900 | 42.0001 | 1.94 | 1.8 | 2.11 | 2.01 | 2.29 | 3.31 |
| *Numida meleagris* | **Nmel** | 1299 | 41.898 | 1.94 | 1.99 | 1.77 | 2.19 | 1.94 | 2.81 |
| *Opisthocomus hoazin* | **Ohoa** | 696 | 42.7 | 2 | 1.99 | 1.9 | 2.32 | 2.39 | 3.53 |
| *Parus major* | **Pmaj** | 16.25 | 41.521 | 2.31 | 2.48 | 2.31 | 2.34 | 2.58 | 3.71 |
| *Passer domesticus* | **Pdom** | 26.51 | 41.183 | 1.87 | 1.93 | 1.81 | 1.87 | 1.78 | 2.82 |
| *Patagioenas fasciata* | **Pfas** | 366.3 | 40.9 | 1.86 | 1.81 | 1.82 | 1.78 | 1.81 | 3.21 |
| *Pelecanus crispus* | **Pecri** | 9512.09 | 41.4 | 1.82 | 1.49 | 1.91 | 1.94 | 2.05 | 3.34 |
| *Phaethon lepturus* | **Plep** | 328.04 | 41.5 | 1.91 | 2.11 | 1.86 | 2.05 | 1.91 | 3.14 |
| *Phalacrocorax carbo* | **Pcar** | 2200 | 41.3 | 1.75 | 1.8 | 1.72 | 1.99 | 1.69 | 2.95 |
| *Phoenicopterus rubber* | **Prub** | 3031.59 | 41.9 | 1.63 | 1.44 | 1.58 | 1.8 | 1.77 | 2.61 |
| *Phylloscopus trochilus* | **Ptro** | 8.7 | 41.2 | 2.26 | 2.68 | 2.12 | 2.15 | 2.14 | 3.39 |
| *Picoides pubescens* | **Ppub** | 28.2 | 44.6 | 3.03 | 2.92 | 3.07 | 3.48 | 2.9 | 4.66 |
| *Podiceps cristatus* | **Pocri** | 730.96 | 41.5 | 1.75 | 1.72 | 1.84 | 1.77 | 1.7 | 2.62 |
| *Pterocles gutturalis* | **Pgut** | 338 | 41.4 | 1.81 | 1.69 | 2.39 | 1.93 | 1.87 | 2.83 |
| *Pygoscelis adeliae* | **Pade** | 4847.67 | 41.8001 | 2 | 1.81 | 2.03 | 2.29 | 2.26 | 3.45 |
| *Struthio camelus* | **Scam** | 111000 | 41.3 | 1.78 | 2.01 | 1.7 | 1.67 | 1.81 | 2.39 |
| *Sturnus vulgaris* | **Svul** | 77.14 | 41.7 | 2.06 | 2.07 | 1.91 | 2.09 | 2.21 | 3.29 |
| *Taeniopygia guttata* | **Tgut** | 12.04 | 41.4526 | 2.26 | 2.52 | 2.08 | 2.48 | 2.27 | 3.85 |
| *Tauraco erythrolophus* | **Tery** | 261.24 | 41.6 | 1.79 | 1.75 | 1.9 | 2.02 | 1.92 | 2.79 |
| *Tinamus major* | **Tmaj** | 1059 | 41.5 | 2.04 | 2.02 | 1.79 | 2.31 | 2.44 | 3.16 |
| *Tyto alba* | **Talb** | 403.32 | 40.2 | 1.85 | 1.67 | 1.98 | 1.99 | 1.84 | 3.42 |
| *Uria lomvia* | **Ulom** | 963.99 | 41.5 | 1.81 | 2.02 | 1.86 | 1.85 | 1.81 | 2.38 |
| *Zosterops lateralis* | **Zlat** | 12.68 | 41.3 | 2.04 | 1.85 | 2.05 | 2.1 | 1.98 | 3.34 |
