# Supplementary material for: A genome-wide investigation of microsatellite mismatches and the association with body mass among bird species
Source: PeerJ. 2018 Mar 14;6:e4495. doi: 10.7717/peerj.4495 (PMC5857172; doi:10.7717/peerj.4495)
Supplement: Table S2 [file peerj-06-4495-s006.docx]

**Table S2** Microsatellite abundance in 65 birds. The paired sample t-test results are shown below the data tables.

| **Species** | **Mono-** | **Di-** | **Tri-** | **Tetra-** | **Penta-** | **Hexa-** | **All** | **Genome**  **Size (bp)** | **Density**  **(counts/Mb)** |
| --- | --- | --- | --- | --- | --- | --- | --- | --- | --- |
| Achl | 24816 | 13709 | 11311 | 19928 | 20246 | 4341 | 94351 | 1044655775 | 90.32 |
| Aros | 115506 | 16551 | 16359 | 32938 | 31834 | 8256 | 221444 | 1121789902 | 197.4 |
| Aaes | 52969 | 11520 | 11261 | 24644 | 20494 | 4540 | 125428 | 1129535839 | 111.04 |
| Apla | 194951 | 25670 | 32238 | 96348 | 55092 | 11741 | 416040 | 1105049323 | 376.49 |
| Abra | 112483 | 33032 | 37492 | 82404 | 53299 | 13007 | 331717 | 1116992394 | 296.97 |
| Acyg | 111464 | 26753 | 30580 | 79349 | 48127 | 10836 | 307109 | 1119151626 | 274.41 |
| Acar | 88395 | 22270 | 19983 | 39050 | 35324 | 7144 | 212166 | 1145149681 | 185.27 |
| Avit | 37673 | 11359 | 11376 | 23234 | 21622 | 4748 | 110012 | 1085711737 | 101.33 |
| Afor | 72873 | 14281 | 18794 | 28393 | 27155 | 4886 | 166382 | 1257483768 | 132.31 |
| Breg | 100616 | 12656 | 16112 | 28841 | 25577 | 4449 | 188251 | 1138861064 | 165.3 |
| Brhi | 52008 | 10250 | 13869 | 21864 | 22520 | 4200 | 124711 | 1105159377 | 112.84 |
| Csqu | 97325 | 23861 | 22200 | 55979 | 46135 | 11338 | 256838 | 1045281893 | 245.71 |
| Cann | 87609 | 21272 | 19029 | 40379 | 40524 | 9610 | 218423 | 1114626264 | 195.96 |
| Ccri | 71052 | 10217 | 11870 | 24497 | 25397 | 4497 | 147530 | 1145705463 | 128.77 |
| Caur | 69648 | 12028 | 14157 | 27192 | 23709 | 4291 | 151025 | 1179513848 | 128.04 |
| Cpel | 65527 | 16240 | 15375 | 39589 | 29262 | 5289 | 171282 | 1125117173 | 152.23 |
| Cvoc | 110312 | 12817 | 14939 | 28987 | 30242 | 6382 | 203679 | 1225129931 | 166.25 |
| Cmac | 114222 | 10991 | 12021 | 27239 | 25147 | 4477 | 194097 | 1095295809 | 177.21 |
| Cstr | 28731 | 13135 | 9741 | 19690 | 20645 | 2910 | 94852 | 1086303309 | 87.32 |
| Cliv | 71058 | 15614 | 16510 | 56514 | 41502 | 7534 | 208732 | 1111581692 | 187.78 |
| Cbra | 47613 | 15066 | 16090 | 26525 | 24586 | 6504 | 136384 | 1095175346 | 124.53 |
| Ccan | 37439 | 11607 | 11267 | 21895 | 21215 | 4182 | 107605 | 1159208195 | 92.83 |
| Egar | 101442 | 14303 | 16777 | 24627 | 25762 | 4576 | 187487 | 1211513874 | 154.75 |
| Ehel | 60777 | 8526 | 9138 | 26725 | 19860 | 2829 | 127855 | 1098400457 | 116.4 |
| Fper | 86888 | 18983 | 16779 | 29331 | 26991 | 5745 | 184717 | 1174046505 | 157.33 |
| Fgla | 80801 | 11094 | 14402 | 25981 | 25793 | 4877 | 162948 | 1153271365 | 141.29 |
| Goki | 51127 | 25462 | 20245 | 27949 | 28297 | 7298 | 160378 | 1114530436 | 143.9 |
| Ggal | 97347 | 24889 | 23284 | 58495 | 39371 | 8023 | 251409 | 1108466630 | 226.81 |
| Gste | 46513 | 9105 | 10685 | 21612 | 20485 | 3539 | 111939 | 1145076370 | 97.76 |
| Gfor | 33721 | 17462 | 20239 | 31628 | 34071 | 8713 | 145834 | 1072484429 | 135.98 |
| Gjap | 97525 | 15085 | 19540 | 31983 | 29477 | 5664 | 199274 | 1265634512 | 157.45 |
| Halb | 72461 | 12143 | 14634 | 27098 | 23960 | 4040 | 154336 | 1144733688 | 134.82 |
| Hleu | 120851 | 18619 | 19652 | 30570 | 28483 | 9329 | 227504 | 1258793725 | 180.73 |
| Lcor | 43589 | 19181 | 18958 | 31557 | 27203 | 6299 | 146787 | 1079577334 | 135.97 |
| Ldis | 94474 | 13669 | 12940 | 26117 | 26108 | 4777 | 178085 | 1148076042 | 155.12 |
| Lstr | 77313 | 18773 | 21623 | 30043 | 33284 | 11732 | 192768 | 1060169706 | 181.83 |
| Mvit | 40724 | 19450 | 16202 | 33089 | 26487 | 5379 | 141331 | 1160253066 | 121.81 |
| Mgal | 36016 | 17547 | 16815 | 44181 | 28053 | 7126 | 149738 | 1061982190 | 141 |
| Mund | 11932 | 13166 | 11559 | 23380 | 18169 | 3437 | 81643 | 1117358947 | 73.07 |
| Mnub | 102471 | 11599 | 13679 | 34158 | 29974 | 5383 | 197264 | 1066568727 | 184.95 |
| Muni | 13366 | 16913 | 16313 | 26857 | 29646 | 7741 | 110836 | 1101046125 | 100.66 |
| Nnot | 50757 | 10191 | 10675 | 25734 | 23429 | 4531 | 125317 | 1062026465 | 118 |
| Nnip | 106490 | 16066 | 17776 | 32315 | 30644 | 5914 | 209205 | 1240309383 | 168.67 |
| Nmel | 74907 | 14477 | 16392 | 33351 | 28243 | 6674 | 174044 | 1043264150 | 166.83 |
| Ohoa | 42171 | 9049 | 10882 | 19842 | 19772 | 3463 | 105179 | 1209082675 | 86.99 |
| Pmaj | 45759 | 21999 | 21770 | 29187 | 30785 | 9674 | 159174 | 1020310768 | 156.01 |
| Pdom | 41330 | 14225 | 17751 | 27993 | 29090 | 6937 | 137326 | 1042720703 | 131.7 |
| Pfas | 61940 | 16740 | 16452 | 46296 | 36225 | 7826 | 185479 | 1089149042 | 170.3 |
| Pecri | 103713 | 11577 | 19463 | 26280 | 26321 | 4532 | 191886 | 1168624213 | 164.2 |
| Plep | 60610 | 9379 | 12497 | 24210 | 22698 | 4514 | 133908 | 1166684827 | 114.78 |
| Pcar | 41336 | 9226 | 12446 | 23702 | 23372 | 4251 | 114333 | 1154672768 | 99.02 |
| Prub | 66636 | 10874 | 13610 | 26561 | 24562 | 3833 | 146076 | 1144756512 | 127.6 |
| Ptro | 94222 | 20091 | 30580 | 53896 | 51582 | 15717 | 266088 | 1066518484 | 249.49 |
| Ppub | 29669 | 19808 | 22405 | 44609 | 120658 | 8831 | 245980 | 1174542329 | 209.43 |
| Pocri | 196839 | 12858 | 13115 | 37953 | 34487 | 5780 | 301032 | 1152104262 | 261.29 |
| Pgut | 113845 | 11517 | 12488 | 24275 | 23769 | 4448 | 190342 | 1079547375 | 176.32 |
| Pade | 66334 | 14315 | 21072 | 27956 | 26550 | 4421 | 160648 | 1226103150 | 131.02 |
| Scam | 96750 | 32488 | 13175 | 32953 | 25430 | 6187 | 206983 | 1257873544 | 164.55 |
| Svul | 64217 | 17277 | 21841 | 34282 | 36622 | 9922 | 184161 | 1036755994 | 177.63 |
| Tgut | 104320 | 21010 | 20834 | 32939 | 35281 | 9664 | 224048 | 1235794146 | 181.3 |
| Tery | 66001 | 11144 | 13801 | 25482 | 24559 | 4857 | 145844 | 1175440150 | 124.08 |
| Tmaj | 106184 | 31577 | 19133 | 48406 | 28573 | 5886 | 239759 | 1059687971 | 226.25 |
| Talb | 171143 | 15053 | 13389 | 38870 | 36174 | 8428 | 283057 | 1138815065 | 248.55 |
| Ulom | 58693 | 15991 | 18290 | 29875 | 30387 | 5732 | 158968 | 1179358714 | 134.79 |
| Zlat | 94400 | 17953 | 21568 | 37146 | 34791 | 9010 | 214868 | 1036003386 | 207.4 |
| Total | 4991894 | 1051753 | 1107443 | 2214973 | 2015132 | 422701 | 11803896 |  |  |

The paired sample t-test results for microsatellite abundance by motif length among the 65 bird species are as follows:

|  | Mono- | | Di- | | Tri- | | Tetra- | | Penta- | | Hexa- | |
| --- | --- | --- | --- | --- | --- | --- | --- | --- | --- | --- | --- | --- |
|  | (76798±37579) | | (16181±5762) | | (17038±5508) | | (34077±14696) | | (31002±13940) | | (6503±2678) | |
|  | t | p | t | p | t | p | t | p | t | p | t | p |
| Mono- |  |  | 13.418 | <0.001 | 13.356 | <0.001 | 10.465 | <0.001 | 9.8173 | <0.001 | 15.413 | <0.001 |
| Di- | 13.418 | <0.001 |  |  | 1.6733 | 0.0992 | 12.55 | <0.001 | 9.5859 | <0.001 | 17.723 | <0.001 |
| Tri- | 13.356 | <0.001 | 1.6733 | 0.0992 |  |  | 12.705 | <0.001 | 9.7818 | <0.001 | 23.804 | <0.001 |
| Tetra- | 10.465 | <0.001 | 12.55 | <0.001 | 12.705 | <0.001 |  |  | 1.92 | 0.0593 | 17.178 | <0.001 |
| Penta- | 9.8173 | <0.001 | 9.5859 | <0.001 | 9.7818 | <0.001 | 1.92 | 0.0593 |  |  | 15.802 | <0.001 |
| Hexa- | 15.413 | <0.001 | 17.723 | <0.001 | 23.804 | <0.001 | 17.178 | <0.001 | 15.802 | <0.001 |  |  |

*Note.* The mean value ± SD are presented in parentheses.
