# Supplementary material for: A genome-wide investigation of microsatellite mismatches and the association with body mass among bird species
Source: PeerJ. 2018 Mar 14;6:e4495. doi: 10.7717/peerj.4495 (PMC5857172; doi:10.7717/peerj.4495)
Supplement: Table S3 [file peerj-06-4495-s007.docx]

**Table S3: Number of imperfect microsatellites in different avian genomes and the percentage of imperfect microsatellites in the corresponding genome.**

| Abbreviated Species names | Imperfect microsatellites | All microsatellites | % of all microsatellites |
| --- | --- | --- | --- |
| Achl | 20420 | 94351 | 0.216426 |
| Aros | 53361 | 221444 | 0.240968 |
| Aaes | 29132 | 125428 | 0.232261 |
| Apla | 96564 | 416040 | 0.232103 |
| Abra | 78385 | 331717 | 0.236301 |
| Acyg | 70469 | 307109 | 0.229459 |
| Acar | 43880 | 212166 | 0.206819 |
| Avit | 23682 | 110012 | 0.215267 |
| Afor | 31941 | 166382 | 0.191974 |
| Breg | 33650 | 188251 | 0.178751 |
| Brhi | 21533 | 124711 | 0.172663 |
| Csqu | 63062 | 256838 | 0.245532 |
| Cann | 56715 | 218423 | 0.259657 |
| Ccri | 28128 | 147530 | 0.19066 |
| Caur | 26162 | 151025 | 0.17323 |
| Cpel | 39589 | 171282 | 0.231133 |
| Cvoc | 39217 | 203679 | 0.192543 |
| Cmac | 36268 | 194097 | 0.186855 |
| Cstr | 17457 | 94852 | 0.184045 |
| Cliv | 54916 | 208732 | 0.263093 |
| Cbra | 31866 | 136384 | 0.233649 |
| Ccan | 22652 | 107605 | 0.210511 |
| Egar | 34586 | 187487 | 0.184471 |
| Ehel | 19308 | 127855 | 0.151015 |
| Fper | 37031 | 184717 | 0.200474 |
| Fgla | 29511 | 162948 | 0.181107 |
| Goki | 32224 | 160378 | 0.200925 |
| Ggal | 52979 | 251409 | 0.210728 |
| Gste | 20539 | 111939 | 0.183484 |
| Gfor | 39515 | 145834 | 0.270959 |
| Gjap | 44790 | 199274 | 0.224766 |
| Halb | 28267 | 154336 | 0.183152 |
| Hleu | 42417 | 227504 | 0.186445 |
| Lcor | 35084 | 146787 | 0.239013 |
| Ldis | 31496 | 178085 | 0.176859 |
| Lstr | 49770 | 192768 | 0.258186 |
| Mvit | 34312 | 141331 | 0.242778 |
| Mgal | 36443 | 149738 | 0.243378 |
| Mund | 20763 | 81643 | 0.254315 |
| Mnub | 38114 | 197264 | 0.193213 |
| Muni | 26284 | 110836 | 0.237143 |
| Nnot | 28588 | 125317 | 0.228125 |
| Nnip | 38731 | 209205 | 0.185134 |
| Nmel | 32604 | 174044 | 0.187332 |
| Ohoa | 21567 | 105179 | 0.20505 |
| Pmaj | 42645 | 159174 | 0.267914 |
| Pdom | 31055 | 137326 | 0.226141 |
| Pfas | 38223 | 185479 | 0.206077 |
| Pecri | 32685 | 191886 | 0.170336 |
| Plep | 23865 | 133908 | 0.178219 |
| Pcar | 21518 | 114333 | 0.188205 |
| Prub | 24235 | 146076 | 0.165907 |
| Ptro | 67288 | 266088 | 0.252879 |
| Ppub | 60001 | 245980 | 0.243926 |
| Pocri | 53435 | 301032 | 0.177506 |
| Pgut | 38926 | 190342 | 0.204506 |
| Pade | 31387 | 160648 | 0.195377 |
| Scam | 33672 | 206983 | 0.16268 |
| Svul | 43457 | 184161 | 0.235973 |
| Tgut | 53580 | 224048 | 0.239145 |
| Tery | 27733 | 145844 | 0.190155 |
| Tmaj | 47004 | 239759 | 0.196047 |
| Talb | 59772 | 283057 | 0.211166 |
| Ulom | 33028 | 158968 | 0.207765 |
| Zlat | 46379 | 214868 | 0.215849 |
