# Supplementary material for: A genome-wide investigation of microsatellite mismatches and the association with body mass among bird species
Source: PeerJ. 2018 Mar 14;6:e4495. doi: 10.7717/peerj.4495 (PMC5857172; doi:10.7717/peerj.4495)
Supplement: Table S4 [file peerj-06-4495-s008.docx]

**Table S4:** Motif length and percentage of imperfection of microsatellites in different species. The paired sample *t*-test results are shown below the data tables.

| **Abbreviated species names** | **Mono-** | **Di-** | **Tri-** | **Tetra-** | **Penta-** | **Hexa-** |
| --- | --- | --- | --- | --- | --- | --- |
| **Achl** | 0.200919 | 0.281202 | 0.260189 | 0.220494 | 0.142942 | 0.310528 |
| **Aros** | 0.233235 | 0.294786 | 0.265175 | 0.248528 | 0.19165 | 0.353319 |
| **Aaes** | 0.227492 | 0.298872 | 0.238167 | 0.229508 | 0.179711 | 0.356388 |
| **Apla** | 0.217014 | 0.274055 | 0.28057 | 0.232293 | 0.216293 | 0.330466 |
| **Abra** | 0.192287 | 0.318207 | 0.304145 | 0.227368 | 0.228091 | 0.303606 |
| **Acyg** | 0.198414 | 0.289126 | 0.292086 | 0.225838 | 0.2173 | 0.305279 |
| **Acar** | 0.199208 | 0.246116 | 0.24701 | 0.18799 | 0.171442 | 0.343925 |
| **Avit** | 0.203913 | 0.256537 | 0.257384 | 0.217655 | 0.161271 | 0.339933 |
| **Afor** | 0.165027 | 0.222393 | 0.307119 | 0.181735 | 0.159676 | 0.301064 |
| **Breg** | 0.167011 | 0.195401 | 0.222753 | 0.180056 | 0.161473 | 0.328388 |
| **Brhi** | 0.142805 | 0.24322 | 0.205566 | 0.195207 | 0.146714 | 0.283333 |
| **Csqu** | 0.230003 | 0.289426 | 0.243829 | 0.219582 | 0.247469 | 0.410037 |
| **Cann** | 0.236688 | 0.330481 | 0.290189 | 0.252631 | 0.224731 | 0.428616 |
| **Ccri** | 0.191099 | 0.198101 | 0.218534 | 0.1819 | 0.161358 | 0.306427 |
| **Caur** | 0.157765 | 0.209594 | 0.240658 | 0.173102 | 0.141128 | 0.278024 |
| **Cpel** | 0.197506 | 0.31367 | 0.297821 | 0.24383 | 0.187205 | 0.348459 |
| **Cvoc** | 0.173698 | 0.236639 | 0.260125 | 0.200228 | 0.171946 | 0.334221 |
| **Cmac** | 0.185962 | 0.194705 | 0.203727 | 0.172987 | 0.166262 | 0.345097 |
| **Cstr** | 0.139362 | 0.262809 | 0.232728 | 0.199746 | 0.142892 | 0.29244 |
| **Cliv** | 0.192955 | 0.307737 | 0.289279 | 0.331405 | 0.230712 | 0.440669 |
| **Cbra** | 0.166026 | 0.323178 | 0.30839 | 0.253007 | 0.191898 | 0.415283 |
| **Ccan** | 0.184754 | 0.283536 | 0.269637 | 0.216259 | 0.161018 | 0.300096 |
| **Egar** | 0.167327 | 0.222051 | 0.293557 | 0.174361 | 0.147077 | 0.312063 |
| **Ehel** | 0.11432 | 0.197865 | 0.207485 | 0.185519 | 0.154481 | 0.265465 |
| **Fper** | 0.182096 | 0.263394 | 0.238095 | 0.195868 | 0.17002 | 0.327241 |
| **Fgla** | 0.163921 | 0.227961 | 0.233648 | 0.18756 | 0.157058 | 0.296904 |
| **Goki** | 0.173744 | 0.232111 | 0.285601 | 0.188236 | 0.130756 | 0.36832 |
| **Ggal** | 0.155803 | 0.296717 | 0.230588 | 0.228122 | 0.225979 | 0.351116 |
| **Gste** | 0.172296 | 0.214937 | 0.238465 | 0.1892 | 0.142934 | 0.283413 |
| **Gfor** | 0.260668 | 0.367312 | 0.276694 | 0.264418 | 0.202107 | 0.397337 |
| **Gjap** | 0.266691 | 0.213656 | 0.225691 | 0.164306 | 0.144553 | 0.288136 |
| **Halb** | 0.168297 | 0.19468 | 0.262608 | 0.175142 | 0.163606 | 0.296782 |
| **Hleu** | 0.147562 | 0.195875 | 0.276511 | 0.182237 | 0.16947 | 0.547218 |
| **Lcor** | 0.200853 | 0.34456 | 0.272233 | 0.250214 | 0.16557 | 0.342753 |
| **Ldis** | 0.155979 | 0.251445 | 0.22187 | 0.186354 | 0.157538 | 0.308143 |
| **Lstr** | 0.221593 | 0.351249 | 0.314202 | 0.262557 | 0.197873 | 0.407092 |
| **Mvit** | 0.213535 | 0.337069 | 0.265893 | 0.246457 | 0.183486 | 0.322922 |
| **Mgal** | 0.25944 | 0.306149 | 0.244484 | 0.22177 | 0.203686 | 0.295257 |
| **Mund** | 0.264331 | 0.344448 | 0.273034 | 0.242216 | 0.18394 | 0.265639 |
| **Mnub** | 0.170155 | 0.194413 | 0.236201 | 0.221705 | 0.1927 | 0.342374 |
| **Muni** | 0.276597 | 0.29232 | 0.273769 | 0.202889 | 0.1723 | 0.338458 |
| **Nnot** | 0.228875 | 0.25081 | 0.241311 | 0.215746 | 0.198941 | 0.358861 |
| **Nnip** | 0.153883 | 0.256193 | 0.279365 | 0.193656 | 0.165644 | 0.326006 |
| **Nmel** | 0.14498 | 0.294467 | 0.243533 | 0.20719 | 0.158659 | 0.314354 |
| **Ohoa** | 0.172346 | 0.275721 | 0.262911 | 0.215099 | 0.171404 | 0.371354 |
| **Pmaj** | 0.254048 | 0.369426 | 0.306752 | 0.248295 | 0.178756 | 0.358177 |
| **Pdom** | 0.236366 | 0.3129 | 0.261675 | 0.214125 | 0.143864 | 0.289895 |
| **Pfas** | 0.133032 | 0.315472 | 0.265925 | 0.221034 | 0.182443 | 0.445311 |
| **Pecri** | 0.131353 | 0.187959 | 0.366388 | 0.169939 | 0.146309 | 0.317299 |
| **Plep** | 0.154595 | 0.236912 | 0.246859 | 0.190624 | 0.141378 | 0.302171 |
| **Pcar** | 0.184706 | 0.227618 | 0.237988 | 0.186102 | 0.141751 | 0.258057 |
| **Prub** | 0.148448 | 0.18742 | 0.209405 | 0.177779 | 0.146649 | 0.295069 |
| **Ptro** | 0.227611 | 0.30984 | 0.285808 | 0.252356 | 0.218332 | 0.382643 |
| **Ppub** | 0.213994 | 0.243387 | 0.251015 | 0.252572 | 0.234564 | 0.411958 |
| **Pocri** | 0.166644 | 0.203686 | 0.21769 | 0.19258 | 0.164294 | 0.377855 |
| **Pgut** | 0.180684 | 0.230702 | 0.318306 | 0.218167 | 0.197779 | 0.388264 |
| **Pade** | 0.162601 | 0.215368 | 0.329869 | 0.183717 | 0.154802 | 0.298801 |
| **Scam** | 0.097953 | 0.308545 | 0.206376 | 0.179802 | 0.149194 | 0.280103 |
| **Svul** | 0.196537 | 0.338369 | 0.301451 | 0.238142 | 0.183332 | 0.355573 |
| **Tgut** | 0.181576 | 0.368396 | 0.292983 | 0.269771 | 0.220714 | 0.426428 |
| **Tery** | 0.159876 | 0.246321 | 0.258532 | 0.208265 | 0.161529 | 0.311303 |
| **Tmaj** | 0.133871 | 0.321722 | 0.229237 | 0.216027 | 0.205299 | 0.326368 |
| **Talb** | 0.1811 | 0.233907 | 0.262977 | 0.245922 | 0.217394 | 0.511747 |
| **Ulom** | 0.194913 | 0.28885 | 0.268617 | 0.193105 | 0.148386 | 0.310188 |
| **Zlat** | 0.18393 | 0.289979 | 0.260154 | 0.223227 | 0.179271 | 0.407325 |

The paired sample t-test results for percentage of imperfection of microsatellites by motif length among the 65 bird species are as follows:

|  | Mono- | | Di- | | Tri- | | Tetra- | | Penta- | | Hexa- | |
| --- | --- | --- | --- | --- | --- | --- | --- | --- | --- | --- | --- | --- |
|  | (0.187±0.039) | | (0.268±0.052) | | (0.262±0.034) | | (0.213±0.032) | | (0.177±0.029) | | (0.342±0.057) | |
|  | t | p | t | p | t | p | t | p | t | p | t | p |
| Mono- |  |  | 13.353 | <0.001 | 13.139 | <0.001 | 5.634 | <0.001 | 2.193 | 0.032 | 19.122 | <0.001 |
| Di- | 13.353 | <0.001 |  |  | 1.010 | 0.3161 | 12.591 | <0.001 | 16.212 | <0.001 | 8.746 | <0.001 |
| Tri- | 13.139 | <0.001 | 1.010 | 0.3161 |  |  | 10.637 | <0.001 | 17.853 | <0.001 | 11.335 | <0.001 |
| Tetra- | 5.634 | <0.001 | 12.591 | <0.001 | 10.637 | <0.001 |  |  | 13.266 | <0.001 | 21.099 | <0.001 |
| Penta- | 2.193 | 0.032 | 16.212 | <0.001 | 17.853 | <0.001 | 13.266 | <0.001 |  |  | 28.003 | <0.001 |
| Hexa- | 19.122 | <0.001 | 8.746 | <0.001 | 11.335 | <0.001 | 21.099 | <0.001 | 28.003 | <0.001 |  |  |

*Note.* The mean value ± SD are presented in parentheses.
