# Supplementary material for: A genome-wide investigation of microsatellite mismatches and the association with body mass among bird species
Source: PeerJ. 2018 Mar 14;6:e4495. doi: 10.7717/peerj.4495 (PMC5857172; doi:10.7717/peerj.4495)
Supplement: Table S5 [file peerj-06-4495-s009.docx]

**Table S5:** Average length of imperfect microsatellites compared to perfect microsatellites.

| **Species** | **perfect microsatellites** | **Imperfect microsatellites** |
| --- | --- | --- |
| **Achl** | 21 | 43 |
| **Aros** | 21 | 41 |
| **Aaes** | 20 | 34 |
| **Apla** | 21 | 36 |
| **Abra** | 21 | 36 |
| **Acyg** | 25 | 48 |
| **Acar** | 20 | 37 |
| **Avit** | 19 | 38 |
| **Afor** | 22 | 39 |
| **Breg** | 19 | 34 |
| **Brhi** | 20 | 37 |
| **Csqu** | 22 | 39 |
| **Cann** | 21 | 46 |
| **Ccri** | 18 | 32 |
| **Caur** | 19 | 31 |
| **Cpel** | 21 | 53 |
| **Cvoc** | 19 | 36 |
| **Cmac** | 18 | 30 |
| **Cstr** | 20 | 37 |
| **Cliv** | 21 | 44 |
| **Cbra** | 21 | 44 |
| **Ccan** | 21 | 41 |
| **Egar** | 19 | 33 |
| **Ehel** | 18 | 33 |
| **Fper** | 19 | 35 |
| **Fgla** | 19 | 33 |
| **Goki** | 19 | 31 |
| **Ggal** | 21 | 38 |
| **Gste** | 19 | 33 |
| **Gfor** | 24 | 50 |
| **Gjap** | 18 | 33 |
| **Halb** | 18 | 31 |
| **Hleu** | 19 | 37 |
| **Lcor** | 19 | 36 |
| **Ldis** | 19 | 34 |
| **Lstr** | 24 | 49 |
| **Mvit** | 20 | 41 |
| **Mgal** | 19 | 33 |
| **Mund** | 18 | 34 |
| **Mnub** | 20 | 36 |
| **Muni** | 20 | 37 |
| **Nnot** | 19 | 33 |
| **Nnip** | 20 | 37 |
| **Nmel** | 20 | 36 |
| **Ohoa** | 20 | 42 |
| **Pmaj** | 21 | 40 |
| **Pdom** | 22 | 35 |
| **Pfas** | 20 | 34 |
| **Pecri** | 19 | 34 |
| **Plep** | 20 | 36 |
| **Pcar** | 20 | 36 |
| **Prub** | 18 | 32 |
| **Ptro** | 23 | 39 |
| **Ppub** | 26 | 56 |
| **Pocri** | 20 | 34 |
| **Pgut** | 19 | 33 |
| **Pade** | 20 | 38 |
| **Scam** | 19 | 33 |
| **Svul** | 22 | 39 |
| **Tgut** | 21 | 44 |
| **Tery** | 19 | 36 |
| **Tmaj** | 20 | 37 |
| **Talb** | 20 | 35 |
| **Ulom** | 18 | 31 |
| **Zlat** | 24 | 39 |
