# Supplementary material for: A genome-wide investigation of microsatellite mismatches and the association with body mass among bird species
Source: PeerJ. 2018 Mar 14;6:e4495. doi: 10.7717/peerj.4495 (PMC5857172; doi:10.7717/peerj.4495)
Supplement: Table S6 [file peerj-06-4495-s010.docx]

**Table S6:** Genomic abundance of microsatellites having a length of 20 bp that either lack mismatch (perfect motifs) or have exactly one mismatch in each locus across species.

|  | **20bp** | |
| --- | --- | --- |
| **Species** | **Mismatches=0** | **Mismatches=1** |
| **Achl** | 3433 | 256 |
| **Aros** | 8858 | 426 |
| **Aaes** | 4327 | 364 |
| **Apla** | 15547 | 1220 |
| **Abra** | 12241 | 1118 |
| **Acyg** | 11924 | 1063 |
| **Acar** | 8204 | 487 |
| **Avit** | 3460 | 366 |
| **Afor** | 6692 | 439 |
| **Breg** | 7880 | 421 |
| **Brhi** | 4549 | 321 |
| **Csqu** | 8951 | 742 |
| **Cann** | 7707 | 621 |
| **Ccri** | 5830 | 341 |
| **Caur** | 6264 | 341 |
| **Cpel** | 6326 | 594 |
| **Cvoc** | 7823 | 466 |
| **Cmac** | 8134 | 381 |
| **Cstr** | 3136 | 287 |
| **Cliv** | 7428 | 597 |
| **Cbra** | 4693 | 337 |
| **Ccan** | 3475 | 279 |
| **Egar** | 6959 | 350 |
| **Ehel** | 4572 | 314 |
| **Fper** | 7642 | 544 |
| **Fgla** | 7098 | 396 |
| **Goki** | 5940 | 378 |
| **Ggal** | 10827 | 767 |
| **Gste** | 3898 | 291 |
| **Gfor** | 4827 | 457 |
| **Gjap** | 7918 | 498 |
| **Halb** | 6324 | 425 |
| **Hleu** | 8383 | 473 |
| **Lcor** | 5042 | 466 |
| **Ldis** | 7263 | 378 |
| **Lstr** | 6293 | 513 |
| **Mvit** | 4912 | 449 |
| **Mgal** | 5590 | 546 |
| **Mund** | 2578 | 397 |
| **Mnub** | 8175 | 423 |
| **Muni** | 3835 | 532 |
| **Nnot** | 4665 | 365 |
| **Nnip** | 7898 | 463 |
| **Nmel** | 6953 | 508 |
| **Ohoa** | 3584 | 305 |
| **Pmaj** | 5221 | 452 |
| **Pdom** | 4749 | 427 |
| **Pfas** | 7143 | 563 |
| **Pecri** | 7677 | 377 |
| **Plep** | 4888 | 319 |
| **Pcar** | 3962 | 402 |
| **Prub** | 5923 | 351 |
| **Ptro** | 9021 | 657 |
| **Ppub** | 8307 | 615 |
| **Pocri** | 13905 | 527 |
| **Pgut** | 8125 | 366 |
| **Pade** | 6010 | 428 |
| **Scam** | 8443 | 449 |
| **Svul** | 6633 | 473 |
| **Tgut** | 7869 | 719 |
| **Tery** | 4974 | 411 |
| **Tmaj** | 9845 | 693 |
| **Talb** | 12665 | 467 |
| **Ulom** | 5337 | 476 |
| **Zlat** | 7467 | 508 |
