# Supplementary material for: A genome-wide investigation of microsatellite mismatches and the association with body mass among bird species
Source: PeerJ. 2018 Mar 14;6:e4495. doi: 10.7717/peerj.4495 (PMC5857172; doi:10.7717/peerj.4495)
Supplement: Table S7 [file peerj-06-4495-s011.docx]

**Table S7:** Number of microsatellites where motif mismatches are either higher or lower than the expected values of mismatches in different bird genomes.

| **Species** | **expected number of mismatches** | **Lower than expected** | **Higher than expected** |
| --- | --- | --- | --- |
| **Achl** | 0.0487794 | 12828 | 7592 |
| **Aros** | 0.0534924 | 34965 | 18396 |
| **Aaes** | 0.0534379 | 19100 | 10032 |
| **Apla** | 0.0501248 | 62115 | 34449 |
| **Abra** | 0.0515275 | 51441 | 26944 |
| **Acyg** | 0.0503536 | 45798 | 24671 |
| **Acar** | 0.0531658 | 28106 | 15774 |
| **Avit** | 0.0529249 | 15824 | 7858 |
| **Afor** | 0.0513621 | 21084 | 10857 |
| **Breg** | 0.051434 | 22703 | 10947 |
| **Brhi** | 0.0502966 | 14863 | 6670 |
| **Csqu** | 0.0531809 | 41792 | 21270 |
| **Cann** | 0.0573447 | 35505 | 21210 |
| **Ccri** | 0.0522661 | 19084 | 9044 |
| **Caur** | 0.0521738 | 18047 | 8115 |
| **Cpel** | 0.0543096 | 24434 | 15155 |
| **Cvoc** | 0.0526788 | 26327 | 12890 |
| **Cmac** | 0.0549184 | 23753 | 12515 |
| **Cstr** | 0.0512316 | 12018 | 5439 |
| **Cliv** | 0.0599002 | 34279 | 20637 |
| **Cbra** | 0.0566459 | 20358 | 11508 |
| **Ccan** | 0.0524886 | 15196 | 7456 |
| **Egar** | 0.0529427 | 24059 | 10527 |
| **Ehel** | 0.0514831 | 13521 | 5787 |
| **Fper** | 0.0547701 | 24177 | 12854 |
| **Fgla** | 0.053542 | 19929 | 9582 |
| **Goki** | 0.057191 | 21201 | 11023 |
| **Ggal** | 0.0521687 | 33260 | 19719 |
| **Gste** | 0.0536955 | 14026 | 6513 |
| **Gfor** | 0.0533333 | 24501 | 14920 |
| **Gjap** | 0.0572832 | 29914 | 14876 |
| **Halb** | 0.0542299 | 19124 | 9143 |
| **Hleu** | 0.0580873 | 28027 | 14390 |
| **Lcor** | 0.0570551 | 22868 | 12216 |
| **Ldis** | 0.0524952 | 20780 | 10716 |
| **Lstr** | 0.048985 | 31459 | 18311 |
| **Mvit** | 0.0544448 | 22002 | 12310 |
| **Mgal** | 0.054138 | 23763 | 12680 |
| **Mund** | 0.0554661 | 13522 | 7241 |
| **Mnub** | 0.0513832 | 25510 | 12604 |
| **Muni** | 0.0565864 | 17195 | 9089 |
| **Nnot** | 0.0547978 | 18758 | 9830 |
| **Nnip** | 0.0520971 | 25974 | 12757 |
| **Nmel** | 0.053823 | 21119 | 11485 |
| **Ohoa** | 0.0481859 | 13909 | 7658 |
| **Pmaj** | 0.0573115 | 26705 | 15940 |
| **Pdom** | 0.0528875 | 20038 | 11017 |
| **Pfas** | 0.0551747 | 24896 | 13327 |
| **Pecri** | 0.0534272 | 22187 | 10498 |
| **Plep** | 0.0521712 | 15935 | 7930 |
| **Pcar** | 0.0485606 | 13987 | 7531 |
| **Prub** | 0.0518054 | 17008 | 7227 |
| **Ptro** | 0.057334 | 42073 | 25215 |
| **Ppub** | 0.0543886 | 39051 | 20950 |
| **Pocri** | 0.0513237 | 35043 | 18392 |
| **Pgut** | 0.0556284 | 25564 | 13362 |
| **Pade** | 0.052512 | 20297 | 11090 |
| **Scam** | 0.0541444 | 22632 | 11040 |
| **Svul** | 0.0526993 | 28082 | 15375 |
| **Tgut** | 0.05157 | 33315 | 20265 |
| **Tery** | 0.0507886 | 18766 | 8967 |
| **Tmaj** | 0.0559393 | 30759 | 16245 |
| **Talb** | 0.0536318 | 38257 | 21515 |
| **Ulom** | 0.0579775 | 21653 | 11375 |
| **Zlat** | 0.0517085 | 29517 | 16862 |
