# Supplementary material for: A genome-wide investigation of microsatellite mismatches and the association with body mass among bird species
Source: PeerJ. 2018 Mar 14;6:e4495. doi: 10.7717/peerj.4495 (PMC5857172; doi:10.7717/peerj.4495)
Supplement: Table S8 [file peerj-06-4495-s012.docx]

**Table S8:** Genomic abundance of imperfect microsatellite loci based on length and number of mismatches.

| **Species** | **≥ 30 bp SSR with ≥ 3 mismatches** | **≥ 30 bp SSR with < 3 mismatches** |
| --- | --- | --- |
| **Achl** | 4115 | 8806 |
| **Aros** | 12078 | 33044 |
| **Aaes** | 5183 | 14559 |
| **Apla** | 17455 | 41909 |
| **Abra** | 14218 | 38891 |
| **Acyg** | 11435 | 28055 |
| **Acar** | 7352 | 19132 |
| **Avit** | 4270 | 8059 |
| **Afor** | 5247 | 14901 |
| **Breg** | 4969 | 12125 |
| **Brhi** | 3406 | 11417 |
| **Csqu** | 13323 | 39016 |
| **Cann** | 14349 | 26301 |
| **Ccri** | 3861 | 8776 |
| **Caur** | 3340 | 8376 |
| **Cpel** | 8847 | 17524 |
| **Cvoc** | 5648 | 14181 |
| **Cmac** | 4950 | 10802 |
| **Cstr** | 2850 | 8139 |
| **Cliv** | 14543 | 21344 |
| **Cbra** | 7827 | 15672 |
| **Ccan** | 4086 | 11319 |
| **Egar** | 4709 | 14697 |
| **Ehel** | 2594 | 5653 |
| **Fper** | 6436 | 14151 |
| **Fgla** | 4293 | 9208 |
| **Goki** | 5399 | 10615 |
| **Ggal** | 10597 | 25266 |
| **Gste** | 3072 | 6670 |
| **Gfor** | 10289 | 22315 |
| **Gjap** | 8907 | 16169 |
| **Halb** | 3912 | 8269 |
| **Hleu** | 8859 | 17719 |
| **Lcor** | 7387 | 14646 |
| **Ldis** | 4590 | 11133 |
| **Lstr** | 13603 | 40298 |
| **Mvit** | 6856 | 11796 |
| **Mgal** | 6274 | 14526 |
| **Mund** | 3809 | 6272 |
| **Mnub** | 5986 | 16186 |
| **Muni** | 5347 | 11191 |
| **Nnot** | 4804 | 9651 |
| **Nnip** | 6322 | 20162 |
| **Nmel** | 6150 | 15494 |
| **Ohoa** | 4255 | 8956 |
| **Pmaj** | 10831 | 22532 |
| **Pdom** | 5921 | 20851 |
| **Pfas** | 7345 | 20866 |
| **Pecri** | 5371 | 12921 |
| **Plep** | 3657 | 9260 |
| **Pcar** | 3110 | 8079 |
| **Prub** | 3141 | 7992 |
| **Ptro** | 17188 | 47103 |
| **Ppub** | 17207 | 51272 |
| **Pocri** | 8086 | 29674 |
| **Pgut** | 6562 | 15657 |
| **Pade** | 5529 | 13686 |
| **Scam** | 5172 | 13920 |
| **Svul** | 9474 | 30059 |
| **Tgut** | 12887 | 31794 |
| **Tery** | 4253 | 9327 |
| **Tmaj** | 8430 | 19023 |
| **Talb** | 10892 | 25227 |
| **Ulom** | 5625 | 12410 |
| **Zlat** | 10171 | 48430 |
