# Supplementary material for: A genome-wide investigation of microsatellite mismatches and the association with body mass among bird species
Source: PeerJ. 2018 Mar 14;6:e4495. doi: 10.7717/peerj.4495 (PMC5857172; doi:10.7717/peerj.4495)
Supplement: Table S9 — Result for the relationship between average mismatches and body mass fitted in PGLS analyses when different parameters were used to search microsatellites in the genomes (minimum score = 15 and mismatch penalty = 3; minimum score = 10 and mismatch penalty = 5). The average mismatches of imperfect microsatellites for the 65 birds are given on the next page below the result table. [file peerj-06-4495-s013.doc]

|  |  |  | **Minimum score =15 and mismatch penalty = 3** | | | | | |  | | **Minimum score = 10 and mismatch penalty = 5** | | | | | |
| --- | --- | --- | --- | --- | --- | --- | --- | --- | --- | --- | --- | --- | --- | --- | --- | --- |
| **Type** |  |  | **Body mass** | | | **GC content** | | |  |  | **Body mass** | | | **GC content** | | |
|  | **Model** | **R^2^** | ***β* ± SE** | ***t*** | **P** | ***β* ± SE** | ***t*** | **P** |  | **R^2^** | ***β* ± SE** | ***t*** | **P** | ***β* ± SE** | ***t*** | **P** |
| All | BM | 0.368 | 0.011 ± 0.002 | 6.061 | <0.001 |  |  |  |  | 0.273 | 0.015 ± 0.003 | 4.86 | <0.001 |  |  |  |
|  | BM+GC | 0.306 | 0.011 ± 0.002 | 4.907 | <0.001 | -1.150 ±0.657 | 1.750 | 0.085 |  | 0.328 | 0.015 ± 0.003 | 4.965 | <0.001 | -2.521 ±1.112 | 2.267 | 0.027 |
| Di | BM | 0.378 | 0.012 ± 0.002 | 6.193 | <0.001 |  |  |  |  | 0.279 | 0.023 ± 0.005 | 4.94 | <0.001 |  |  |  |
|  | BM+GC | 0.408 | 0.012 ± 0.002 | 6.253 | <0.001 | -1.258 ±0.719 | 1.748 | 0.085 |  | 0.33 | 0.023 ± 0.004 | 5.042 | < 0.001 | -2.864 ±1.268 | 2.259 | 0.027 |
| Tri | BM | 0.112 | 0.006 ± 0.002 | 2.824 | 0.006 |  |  |  |  | 0.27 | 0.016 ± 0.003 | 4.828 | <0.001 |  |  |  |
|  | BM+GC | 0.114 | 0.006 ± 0.002 | 2.785 | 0.007 | -0.300 ±0.658 | 0.456 | 0.650 |  | 0.307 | 0.016 ± 0.003 | 4.873 | < 0.001 | -2.091 ±1.155 | 1.810 | 0.075 |
| Tetra | BM | 0.421 | 0.013 ±0.002 | 6.767 | < 0.001 |  |  |  |  | 0.288 | 0.019 ±0.004 | 5.054 | < 0.001 |  |  |  |
|  | BM+GC | 0.426 | 0.013 ±0.002 | 6.723 | < 0.001 | -0.487±0.683 | 0.713 | 0.478 |  | 0.39 | 0.019 ±0.004 | 5.343 | < 0.001 | -4.174±1.298 | 3.217 | 0.002 |
| Penta | BM | 0.226 | 0.011 ±0.002 | 4.29 | < 0.001 |  |  |  |  | 0.125 | 0.011 ±0.004 | 3.001 | 0.004 |  |  |  |
|  | BM+GC | 0.241 | 0.011 ±0.002 | 4.271 | < 0.001 | -0.973 ±0.896 | 1.086 | 0.282 |  | 0.196 | 0.011 ±0.004 | 3.054 | 0.003 | -3.121±1.330 | 2.346 | 0.022 |
| Hexa | BM | 0.254 | 0.010 ±0.002 | 4.627 | < 0.001 |  |  |  |  | 0.229 | 0.012 ±0.003 | 4.324 | < 0.001 |  |  |  |
|  | BM+GC | 0.255 | 0.010 ±0.002 | 4.586 | < 0.001 | -0.304 ±0.819 | 0.372 | 0.712 |  | 0.236 | 0.012 ±0.003 | 4.293 | < 0.001 | -0.800 ±1.021 | 0.784 | 0.436 |

**Table S9: Result for the relationship between average mismatches and body mass fitted in PGLS analyses when different parameters were used to search microsatellites in the genomes (minimum score = 15 and mismatch penalty = 3; minimum score = 10 and mismatch penalty = 5). The average mismatches of imperfect microsatellites for the 65 birds are given on the next page below the result table.**

**The average mismatches of imperfect microsatellites when different parameters were used to search microsatellites in the genomes**.

|  | **Minimum score =15 and mismatch penalty = 3** | | | | | |  | **Minimum score = 10 and mismatch penalty = 5** | | | | | |
| --- | --- | --- | --- | --- | --- | --- | --- | --- | --- | --- | --- | --- | --- |
|  | **All** | **Di-** | **Tri-** | **Tetra-** | **Penta-** | **Hexa-** |  | **All** | **Di-** | **Tri-** | **Tetra-** | **Penta-** | **Hexa-** |
| **Achl** | 3.06 | 3.16 | 3.07 | 3.22 | 3.22 | 5.74 |  | 2.07 | 1.91 | 1.96 | 2.14 | 2.13 | 4.11 |
| **Aros** | 3.03 | 3.52 | 2.82 | 3.76 | 3.43 | 4.74 |  | 2.21 | 2.26 | 2.01 | 2.55 | 2.32 | 3.54 |
| **Aaes** | 2.77 | 2.88 | 2.62 | 3.13 | 2.92 | 3.69 |  | 1.80 | 1.85 | 1.67 | 1.86 | 1.92 | 2.91 |
| **Apla** | 2.78 | 2.86 | 3.02 | 2.95 | 2.93 | 3.67 |  | 1.79 | 1.79 | 1.80 | 1.65 | 1.70 | 2.83 |
| **Abra** | 2.77 | 3.06 | 2.92 | 2.89 | 2.81 | 3.34 |  | 1.83 | 2.07 | 1.78 | 1.71 | 1.93 | 2.65 |
| **Acyg** | 3.32 | 2.99 | 3.13 | 3.29 | 5.03 | 6.04 |  | 2.41 | 2.11 | 1.90 | 2.19 | 3.93 | 5.85 |
| **Acar** | 2.81 | 2.87 | 3.36 | 2.85 | 2.89 | 4.63 |  | 1.96 | 1.88 | 2.08 | 1.90 | 1.91 | 3.88 |
| **Avit** | 2.81 | 3.04 | 3.04 | 3.03 | 2.87 | 4.73 |  | 2.02 | 1.87 | 2.00 | 2.34 | 2.01 | 3.80 |
| **Afor** | 2.69 | 3.01 | 2.98 | 2.85 | 3.08 | 3.92 |  | 1.99 | 1.75 | 2.05 | 2.15 | 2.37 | 2.99 |
| **Breg** | 2.5 | 2.91 | 2.55 | 2.7 | 2.74 | 3.87 |  | 1.75 | 1.80 | 1.67 | 1.76 | 1.88 | 2.95 |
| **Brhi** | 2.54 | 2.77 | 2.62 | 2.85 | 2.57 | 3.85 |  | 1.86 | 1.83 | 1.70 | 2.23 | 1.92 | 3.07 |
| **Csqu** | 2.93 | 3.07 | 2.84 | 3.1 | 3.51 | 5.30 |  | 2.09 | 2.12 | 1.86 | 1.99 | 2.33 | 4.34 |
| **Cann** | 3.78 | 4.42 | 4.16 | 4.06 | 4.05 | 6.76 |  | 2.66 | 2.83 | 2.56 | 3.06 | 2.75 | 4.50 |
| **Ccri** | 2.51 | 2.64 | 2.66 | 2.82 | 2.75 | 3.59 |  | 1.68 | 1.49 | 1.57 | 1.83 | 1.87 | 2.79 |
| **Caur** | 2.5 | 2.68 | 3.09 | 2.66 | 2.56 | 3.48 |  | 1.64 | 1.55 | 1.72 | 1.78 | 1.71 | 2.60 |
| **Cpel** | 3.64 | 3.82 | 3.6 | 4.41 | 3.85 | 5.59 |  | 2.88 | 2.90 | 2.58 | 3.90 | 2.95 | 4.29 |
| **Cvoc** | 2.64 | 2.99 | 2.99 | 3.03 | 2.97 | 4.73 |  | 1.87 | 1.91 | 2.00 | 2.07 | 2.07 | 3.69 |
| **Cmac** | 2.45 | 2.57 | 2.6 | 2.9 | 2.54 | 3.85 |  | 1.66 | 1.49 | 1.56 | 1.83 | 1.66 | 2.88 |
| **Cstr** | 2.75 | 2.91 | 2.99 | 2.85 | 2.98 | 4.61 |  | 1.92 | 1.94 | 1.79 | 2.10 | 1.92 | 3.71 |
| **Cliv** | 3.79 | 3.13 | 3.75 | 4.99 | 3.72 | 6.54 |  | 2.61 | 2.08 | 2.33 | 3.43 | 2.18 | 4.46 |
| **Cbra** | 3.34 | 3.29 | 3.24 | 3.77 | 3.57 | 6.63 |  | 2.50 | 2.16 | 2.13 | 2.99 | 2.62 | 4.48 |
| **Ccan** | 3.01 | 3.56 | 3.3 | 3.17 | 3.34 | 4.63 |  | 2.13 | 2.30 | 2.13 | 2.29 | 2.46 | 3.35 |
| **Egar** | 2.44 | 2.74 | 2.64 | 2.79 | 2.66 | 4.27 |  | 1.74 | 1.63 | 1.66 | 2.01 | 2.01 | 3.28 |
| **Ehel** | 2.52 | 2.75 | 2.72 | 2.85 | 2.78 | 3.42 |  | 1.70 | 1.63 | 1.63 | 1.94 | 1.82 | 2.76 |
| **Fper** | 2.79 | 3.28 | 2.86 | 3.12 | 3.08 | 4.02 |  | 1.93 | 2.03 | 1.77 | 2.15 | 2.15 | 3.35 |
| **Fgla** | 2.51 | 2.71 | 2.6 | 2.75 | 2.75 | 3.72 |  | 1.73 | 1.57 | 1.66 | 1.90 | 1.88 | 2.74 |
| **Goki** | 2.37 | 2.43 | 2.64 | 2.42 | 2.16 | 3.17 |  | 1.75 | 1.62 | 2.07 | 1.78 | 1.67 | 2.49 |
| **Ggal** | 3.04 | 3.34 | 2.83 | 3.17 | 3.48 | 4.30 |  | 1.99 | 2.01 | 1.61 | 2.07 | 2.02 | 3.47 |
| **Gste** | 2.57 | 2.85 | 3.11 | 2.74 | 2.64 | 3.95 |  | 1.77 | 1.75 | 2.09 | 1.93 | 1.77 | 3.13 |
| **Gfor** | 3.91 | 4.5 | 3.39 | 4.58 | 4.03 | 6.56 |  | 2.74 | 2.45 | 2.33 | 3.42 | 2.54 | 4.68 |
| **Gjap** | 2.6 | 2.55 | 2.48 | 2.54 | 2.48 | 3.13 |  | 1.90 | 1.72 | 1.63 | 1.64 | 1.77 | 2.35 |
| **Halb** | 2.52 | 2.56 | 3.14 | 2.62 | 2.57 | 3.84 |  | 1.66 | 1.43 | 1.85 | 1.74 | 1.77 | 2.74 |
| **Hleu** | 2.76 | 2.7 | 3.16 | 2.71 | 2.62 | 7.20 |  | 2.16 | 1.55 | 1.93 | 1.80 | 1.89 | 5.26 |
| **Lcor** | 3 | 3.69 | 2.78 | 3.41 | 2.85 | 4.08 |  | 2.04 | 2.19 | 1.82 | 2.29 | 2.05 | 3.29 |
| **Ldis** | 2.52 | 2.82 | 2.7 | 2.89 | 2.63 | 3.93 |  | 1.78 | 1.73 | 1.69 | 2.17 | 1.86 | 3.15 |
| **Lstr** | 3.31 | 3.84 | 3.37 | 3.69 | 3.38 | 5.77 |  | 2.39 | 2.48 | 2.41 | 2.56 | 2.28 | 4.26 |
| **Mvit** | 3.3 | 3.92 | 3.34 | 3.47 | 3.58 | 4.86 |  | 2.20 | 2.28 | 2.24 | 2.28 | 2.39 | 3.88 |
| **Mgal** | 2.78 | 3.06 | 2.92 | 2.84 | 2.59 | 3.09 |  | 1.78 | 1.95 | 1.67 | 1.64 | 1.76 | 2.41 |
| **Mund** | 2.93 | 3.25 | 2.82 | 3.02 | 2.95 | 3.96 |  | 1.85 | 1.82 | 1.76 | 1.84 | 1.98 | 3.13 |
| **Mnub** | 2.84 | 2.94 | 3.04 | 3.47 | 3.29 | 4.32 |  | 1.86 | 1.91 | 1.97 | 2.06 | 2.01 | 3.46 |
| **Muni** | 3.09 | 3.31 | 3.02 | 3.13 | 2.95 | 4.54 |  | 2.07 | 2.04 | 1.95 | 2.02 | 1.94 | 3.32 |
| **Nnot** | 2.78 | 2.84 | 2.79 | 3.1 | 3.05 | 4.11 |  | 1.83 | 1.61 | 1.66 | 2.07 | 1.98 | 2.93 |
| **Nnip** | 2.72 | 3.03 | 3.03 | 2.96 | 3.17 | 4.39 |  | 1.95 | 1.78 | 2.09 | 2.02 | 2.35 | 3.50 |
| **Nmel** | 2.7 | 3.06 | 2.71 | 2.96 | 2.62 | 3.79 |  | 1.93 | 1.97 | 1.75 | 2.18 | 1.94 | 2.91 |
| **Ohoa** | 2.94 | 3.24 | 3.08 | 3.16 | 3.72 | 5.44 |  | 2.02 | 1.98 | 1.89 | 2.30 | 2.40 | 3.64 |
| **Pmaj** | 3.3 | 4.31 | 3.3 | 3.29 | 3.44 | 4.98 |  | 2.30 | 2.45 | 2.31 | 2.33 | 2.59 | 3.80 |
| **Pdom** | 2.8 | 2.94 | 2.67 | 3.09 | 2.7 | 3.67 |  | 1.87 | 1.92 | 1.81 | 1.86 | 1.77 | 2.90 |
| **Pfas** | 2.93 | 2.7 | 3.11 | 3.28 | 2.93 | 4.46 |  | 1.86 | 1.80 | 1.81 | 1.77 | 1.80 | 3.29 |
| **Pecri** | 2.66 | 2.62 | 3.65 | 2.75 | 2.97 | 4.62 |  | 1.79 | 1.50 | 1.89 | 1.94 | 2.05 | 3.56 |
| **Plep** | 2.69 | 3.25 | 2.82 | 2.96 | 2.89 | 4.04 |  | 1.88 | 2.13 | 1.86 | 2.04 | 1.95 | 3.32 |
| **Pcar** | 2.67 | 3.04 | 2.94 | 2.91 | 2.65 | 3.71 |  | 1.77 | 1.79 | 1.71 | 2.01 | 1.71 | 3.09 |
| **Prub** | 2.43 | 2.57 | 2.53 | 2.64 | 2.66 | 3.80 |  | 1.65 | 1.41 | 1.56 | 1.81 | 1.80 | 2.75 |
| **Ptro** | 3.49 | 3.91 | 3.17 | 3.94 | 3.66 | 5.04 |  | 2.25 | 2.66 | 2.11 | 2.14 | 2.13 | 3.48 |
| **Ppub** | 4.32 | 4.33 | 4.02 | 4.28 | 4.78 | 6.65 |  | 3.02 | 2.91 | 3.06 | 3.50 | 2.91 | 4.74 |
| **Pocri** | 2.58 | 2.62 | 2.91 | 3.06 | 2.88 | 3.95 |  | 1.73 | 1.73 | 1.81 | 1.72 | 1.69 | 2.79 |
| **Pgut** | 2.67 | 2.67 | 3.58 | 3 | 2.87 | 4.64 |  | 1.81 | 1.71 | 2.39 | 1.91 | 1.86 | 2.96 |
| **Pade** | 2.8 | 3.2 | 3.07 | 2.98 | 3.27 | 4.16 |  | 2.00 | 1.81 | 2.02 | 2.31 | 2.28 | 3.77 |
| **Scam** | 2.54 | 3.02 | 2.65 | 2.6 | 2.51 | 3.29 |  | 1.79 | 2.01 | 1.70 | 1.65 | 1.79 | 2.53 |
| **Svul** | 3.05 | 3.26 | 2.89 | 3.4 | 3.25 | 4.64 |  | 2.05 | 2.06 | 1.90 | 2.08 | 2.21 | 3.34 |
| **Tgut** | 3.34 | 4.42 | 3.21 | 3.68 | 3.63 | 5.71 |  | 2.25 | 2.49 | 2.09 | 2.49 | 2.27 | 3.96 |
| **Tery** | 2.65 | 2.93 | 3.17 | 2.9 | 2.72 | 3.91 |  | 1.80 | 1.73 | 1.89 | 1.98 | 1.95 | 2.89 |
| **Tmaj** | 2.89 | 3.14 | 2.74 | 3.07 | 3.31 | 4.27 |  | 2.06 | 2.01 | 1.78 | 2.32 | 2.46 | 3.46 |
| **Talb** | 2.96 | 2.71 | 3.33 | 3.71 | 3.14 | 6.59 |  | 1.85 | 1.66 | 1.95 | 1.96 | 1.84 | 3.62 |
| **Ulom** | 2.6 | 3.08 | 2.69 | 2.78 | 2.46 | 3.49 |  | 1.80 | 1.99 | 1.85 | 1.84 | 1.81 | 2.53 |
| **Zlat** | 3.01 | 2.91 | 3 | 3.39 | 3.21 | 4.60 |  | 2.03 | 1.84 | 2.04 | 2.10 | 1.97 | 3.42 |
